# Prevalence and appropriateness of omeprazole prescription in dogs at a veterinary teaching hospital before and after the publication of the ACVIM consensus statement on the rational administration of gastrointestinal protectants

**DOI:** 10.3389/fvets.2024.1352496

**Published:** 2024-05-07

**Authors:** Ángel Sainz, Mercedes García-Sancho, Alejandra Villaescusa, Fernando Rodríguez-Franco, David Díaz-Regañón, Patricia Olmeda, Stanley L. Marks

**Affiliations:** ^1^Department of Animal Medicine and Surgery, College of Veterinary Medicine, Complutense University of Madrid, Madrid, Spain; ^2^Department of Medicine and Epidemiology, School of Veterinary Medicine, University of California, Davis, CA, United States

**Keywords:** acid, canine, gastroesophageal reflux, proton pump inhibitor, ulcer, erosion, NSAID

## Abstract

**Introduction:**

Overprescribing of acid suppressants is a common phenomenon in human and small animal patients, leading to potential deleterious gastrointestinal (GI) and non-GI consequences. The impact of consensus statements on veterinary prescribing habits in clinical practice have not been fully evaluated. This study aimed to compare the prescribing habits of the proton pump inhibitor (PPI), omeprazole, in dogs in an academic veterinary teaching hospital before and after the publication of the American College of Veterinary Internal Medicine (ACVIM) consensus statement on rational use of gastrointestinal protectants.

**Methods:**

Evaluation of the prescribing habits of omeprazole in dogs during the years 2017 and 2021 was retrospectively compared. These years were selected to reflect a 12-month period prior to and following the publication of the consensus statement. One hundred dogs from each year were randomly selected. Dose, frequency of administration, duration of treatment, concurrent prescription of more than one gastroprotectant and indications for prescribing omeprazole were analyzed.

**Results:**

A significant increase in the cases that received omeprazole q12h (*p* < 0.0001) or that underwent a tapering dose after ≥4 week-therapy (*p* > 0.0001) was detected after the publication of the 2018 ACVIM consensus statement. Considering the indications, there was also a significant increase in the appropriate prescription of omeprazole in the second compared to the first period of study (*p* < 0.0001). Fifteen of 16 clinicians (94%) involved in the prescription of omeprazole indicated that their reading of the consensus statement had changed their clinical practice regarding PPI administration in dogs.

**Discussion:**

These results support the beneficial impact of the ACVIM consensus statement on the judicious prescribing of omeprazole in an academic veterinary hospital. These results should not be extrapolated to first-opinion veterinary practices, and further efforts should be made to ensure that PPIs are prescribed prudently with a clear indication and regular review of the appropriateness of continued administration to minimize possible risks and adverse drug interactions.

## 1 Introduction

Antacids, histamine type-2 receptor antagonists, proton-pump inhibitors (PPIs), misoprostol, and sucralfate are gastroprotectants widely used in both human and veterinary medicine (1, 2). Omeprazole was the first commercially available PPI to inhibit the apical H^+^/K^+^-ATPase pump on the parietal cell (3). Since then, other similar PPIs such as lansoprazole, pantoprazole, esomeprazole, rabeprazole and ilaprazole have been marketed, and there has been a substantial, sustained, and inexplicable increase in the prescription of PPIs in human medicine (4). This has resulted in PPIs being one of the most frequently prescribed class of drugs in human medicine in different latitudes including Europe, United States and China (1, 4–6). This increase in acid suppressant utilization has been commensurate with the inappropriate prescription of this class of drugs in about 50% of hospitalized human patients and outpatient settings, with the prevalence being higher in older patients (7, 8).

Gastroprotectants are also widely used by veterinarians, but the number of studies assessing their efficacy in dogs and cats is still limited, as most studies have been conducted in healthy animals (9–12). It has been well documented that adequate suppression of acid secretion improves healing in dogs with gastroduodenal ulceration and erosion (GUE) (2, 11, 13, 14). PPIs have also shown efficacy for the management of gastroesophageal reflux and esophagitis in dogs (15–17) and in prevention of exercise-induced gastritis in working dogs (14).

Studies evaluating the prescribing habits of small animal veterinary practitioners related to gastroprotectants have mainly evaluated individual specialty and referral hospitals in the United States and United Kingdom (UK) (18–21). A longitudinal analysis of electronic health records from first opinion veterinary practices in the UK showed that 37.7% of dogs with acute diarrhea were prescribed gastrointestinal agents (22), including gastroprotectants. It is estimated that around 40% of dogs admitted for hospitalization to veterinary hospitals are prescribed a PPI, with omeprazole being the most commonly recommended gastroprotectant (20, 21). Similar to the situation in human medicine, inappropriate prescription of acid suppressants is common in small animal veterinary medicine (2, 18, 19, 21). Omeprazole is also often administered outside dosing recommendations, reducing efficacy or increasing the risks of adverse effects depending on the dose implemented (20).

An American College of Veterinary Internal Medicine (ACVIM) consensus statement was published in 2018 to advocate for the rational administration of gastrointestinal protectants in dogs and cats (2). ACVIM consensus statements seek to provide the veterinary community with up-to-date information on the pathophysiology, diagnosis, and treatment of clinically important animal diseases (2). There has been no evaluation of the global implementation of the ACVIM consensus statement recommendations in small animal veterinary medicine to date or a comparison of prescribing practices before and after the publication of the ACVIM consensus statement (18, 23, 24).

The aim of this study was to describe and compare the prescribing habits of veterinarians in an academic veterinary teaching hospital related to use of omeprazole in dogs before and after the publication of the 2018 ACVIM consensus statement on rational use of gastrointestinal protectants.

## 2 Materials and methods

### 2.1 Study design

This retrospective observational study aimed to assess the prescription patterns of veterinarians related to use of omeprazole in dogs at the Complutense Veterinary Teaching Hospital, a multidisciplinary referral veterinary medicine teaching hospital in Madrid. This hospital treats emergencies, first-opinion and mainly referral cases from primary care veterinarians in the surrounding area.

### 2.2 Participants, exclusion and inclusion criteria, and sampling

Electronic medical records from dogs prescribed omeprazole by hospital veterinarians during 2 years, 2017 and 2021, were retrieved from the hospital management software program. Exclusion criteria comprised cases already prescribed omeprazole by referring veterinarians, those administered a different proton pump inhibitor, and dogs receiving omeprazole for <24 h. Cases with incomplete data in electronic medical records were also excluded from analysis.

Considering the global data of omeprazole prescriptions in dogs during the study years at the Complutense Veterinary Teaching Hospital, the sample size was calculated using G*Power software, based on an expected improvement of 75% in prescribing practices for the use of omeprazole (18). Using an alpha of 0.05 and a beta of 0.2 (power 80%), 100 dogs per group were required for a *p* < 0.05. For this purpose, all cases that met the inclusion criteria were sorted alphabetically by the dog’s name, and data were obtained from the medical records of the first 100 cases listed in 2017 and in 2021, respectively.

### 2.3 Data collection

Signalment (age, sex and breed), omeprazole dose (correct versus incorrect; doses <0.7 mg/kg were considered incorrect), frequency of administration (q12h or 24h), treatment duration (number of days), presence of dose tapering in dogs managed with omeprazole for >4 weeks (yes or no), concurrent use of other gastroprotectants, and indications for prescription were obtained from medical records.

### 2.4 Evaluation of appropriateness

The ACVIM consensus statement (2) guided the classification of the appropriateness of omeprazole administration based on different clinical scenarios such as GUE, non-erosive gastritis, hepatic disease, renal disease, pancreatitis, reflux esophagitis, *Helicobacter pylori* infection, thrombocytopenia induced-bleeding, prophylaxis for non-steroidal anti-inflammatory drugs (NSAIDs) or glucocorticoid treatment, concomitant use of clopidogrel, or other indications. Based on current scientific evidence, indications were classified into categories with proven benefit, equivocal benefit, and inappropriate use (2, 18–20, 25–28). Briefly, prescription of omeprazole was considered appropriate for GUE associated with primary gastrointestinal (GI) disease (inflammatory bowel disease, low-grade intestinal lymphoma, ulcerated GI tumor with hemorrhage, erosions due to GI foreign bodies, or NSAID or glucocorticoid-associated GUE) or secondary to hepatic disease, portal hypertension, renal disease, and pancreatitis. In addition, administration of omeprazole was deemed appropriate for the management of esophagitis secondary to gastroesophageal reflux or regurgitation during anesthesia and in working or sport dogs exhibiting evidence of GUE during stressful events. A second category of equivocal benefit was reserved for disorders in which the benefits of administration of omeprazole was unproven or controversial, e.g., thrombocytopenia or coagulopathy-induced GI bleeding, pre- or post-coil embolization for intrahepatic portosystemic shunts, gastric, intestinal, or pancreatic neoplasia with no evidence of GUE, chronic kidney disease (CKD) International Renal Interest Society (IRIS) stage 4 with no evidence of GUE, and for management of dogs with hydrocephalus. Acid suppressant therapy with omeprazole was deemed inappropriate for the following diseases or scenarios in the absence of evidence for GUE or esophagitis: renal disease (acute kidney injury (AKI) or CKD IRIS stage 1, 2, or 3), pancreatitis, GI foreign body, dietary indiscretion, GI surgery, inflammatory bowel disease or low grade intestinal lymphoma, non-specific GI signs of inappetence, vomiting, weight loss, diarrhea of unknown etiology, and hepatic disease with no evidence of concurrent portal hypertension.

Evaluation Periods: The evaluation periods (2017 and 2021) were chosen in consideration of the publication of the ACVIM consensus statement on this topic in 2018 (2). No significant legal provisions regarding the prescription of gastroprotectants for dogs and potentially affecting the results occurred during the study period.

### 2.5 Questionnaire to clinicians

An anonymous brief questionnaire comprising two questions was sent to the clinicians who managed dogs enrolled in the study. No demographic data were included to preserve privacy and confidentiality. Clinicians were asked whether they had fully read the ACVIM consensus statement (2), and if their reading of the statement had modified their prescription practice of PPIs in dogs. The studies involving human participants were reviewed and approved by the Institutional Review Board of the Complutense Veterinary Teaching Hospital.

### 2.6 Statistical analysis

The data obtained were statistically analyzed using a statistical software program (SAS, version 9.4, SAS Institute, Cary, NC, United States). The Shapiro–Wilk test was performed to assess the normal distribution of dog age and omeprazole dose. Comparison of age and dose between years (2017 and 2021) was performed using a Student *T*-test. Other demographic data (sex and breed) and variables related to omeprazole prescription were analyzed using the Fisher’s exact and Chi-squared tests. The level of significance level was set at *p* < 0.05.

## 3 Results

### 3.1 Study population and global omeprazole prescription

In 2017, 589 (10.9%) of the 5,384 dogs evaluated at the Complutense Veterinary Teaching Hospital were prescribed omeprazole, while 551 of the 5,801 (9.5%) dogs evaluated in 2021 were prescribed this drug, reflecting a significant decrease in the prescription of omeprazole over time (*p* = 0.01). 113 cases from 2017 and 2021 were reviewed in order to obtain 100 cases for data analysis as 13 cases from each year had to be excluded due to incomplete data in the electronic record.

The mean age of the dogs included in the study was 10.4 ± 3.5 years in 2017 (range 3 to 17 years) and 13.8 ± 3.2 years in 2021 (range 7 to 21 years) (*p* < 0.0001). Thirty-eight female dogs and 62 male dogs were included in 2017, whereas 42 female dogs and 58 male dogs were included in 2021. No significant differences were found for sex (*p* = 0.56). There were 19 mixed-breed and 81 purebred dogs enrolled in 2017, and 26 mixed-breed and 74 purebred dogs enrolled in 2021. There was no significant difference between mixed breed versus purebred dogs during the 2 years evaluated (*p* = 0.31). The most common breeds enrolled in 2017 were the Labrador Retriever (*n* = 11), German Shepherd (*n* = 8), Beagle (*n* = 5), and Golden Retriever (*n* = 5), and in 2021, the Labrador Retriever (*n* = 6), West Highland White Terrier (*n* = 6), and Beagle (*n* = 5). There were 6 brachycephalic dogs enrolled in 2017 and 7 brachycephalic dogs enrolled in 2021. The mean body weight of the dogs evaluated in 2017 was 24.4 ± 13.4kg and 23.8 ± 14.9kg in 2021 (*p* = 0.76).

### 3.2 Dose, frequency, and tapering of omeprazole administration

Therapeutic doses (always higher than 0.7 mg/kg) were used in all dogs prescribed omeprazole in both 2017 and 2021, with no significant differences between the two study periods (*p* = 1.0). No dog received more than 1.1 mg/kg dose. Omeprazole was administered q12h to 5% of the dogs that were prescribed the drug in 2017 and q12h to 84% of the dogs that were prescribed the drug in 2021 (*p* < 0.0001). In addition, there was a significant increase in the number of dogs that underwent a tapering of omeprazole following administration of the drug for ≥4 weeks in 2021 (*n* = 34 dogs) compared to 2017 (*n* = 6 dogs) (*p* < 0.0001). An additional 5 owners from 2021 started to taper omeprazole in their dogs after extended administration but aborted due to subjective worsening of clinical signs in the first few days. Three dogs (two in 2017 and one in 2021) were concurrently treated with famotidine.

### 3.3 Indications

Omeprazole was appropriately prescribed in 37% of dogs evaluated in 2017 and in 74% of the dogs evaluated in 2021 according to the ACVIM consensus statement guidelines (2) (*p* < 0.0001) (Table 1). Appropriate indications for prescription of omeprazole in 2017 (*n* = 37) included 32 dogs with GUE: 30 dogs had GUE due to primary GI disease, one dog had pancreatitis, and one dog had portal hypertension. Furthermore, 5 dogs with esophagitis were prescribed omeprazole. Inappropriate indications for prescription of omeprazole in 2017 included 38 dogs in which the drug was administered for prophylaxis of NSAID/glucocorticoid administration without evidence of GUE, 17 dogs with non-erosive gastritis, 3 dogs with liver disease and no evidence of portal hypertension or GUE, 3 dogs with renal disease (2 with CKD IRIS II, and 1 with AKI) without concurrent evidence of GUE, and 2 dogs with pancreatitis without evidence of GUE. There were no dogs that received omeprazole for an equivocal reason in 2017.

**Table 1:** Indications for omeprazole prescription in 200 dogs at a veterinary teaching hospital during the years 2017 and 2021.

Omeprazole prescription	Year 2017 (%) *N* = 100	Year 2021 (%) *N* = 100	*p*-value
GI-associated GUE	30	53	0.0010
Non-specific gastritis	17	5	0.0067
Liver disease	4	6	0.52
Liver disease with portal hypertension	1	5	0.10
Without GUE	3	0	0.08
With GUE	0	1	0.32
Renal disease	3	8	0.12
Without GUE	3^a^	1^b^	0.31
With GUE	0	7^c^	0.007
Pancreatitis	3	5	0.72
Without GUE	2	1	0.56
With GUE	1	4	0.18
Esophagitis (post-anesthesia)	5	4	0.73
Thrombocytopenia	0	1	1.0000
Prophylaxis (NSAIDs or steroids)	38	16	0.0005
Clopidogrel concurrent	0	0	1.0000
*Helicobacter* spp.	0	0	1.0000
Hydrocephalus	0	2	0.16

a 2 dogs with chronic kidney disease (CKD) IRIS II and 1 dog with acute kidney injury (AKI), without GUE.

b 1 dog with CKD IRIS II, without GUE.

c 3 dogs with CKD IRIS II, 2 with CKD IRIS III, and 2 with CKD IRIS IV, with GUE.

GI, gastrointestinal; GUE, gastroduodenal ulceration or erosion; IRIS, international renal interest society; NSAID, non-steroidal anti-inflammatory drug.

Appropriate indications for prescription of omeprazole during the second study period (2021) were documented in 74 dogs. Fifty-three had GUE associated with primary GI disease, 4 had esophagitis, and 17 had GUE secondary to different diseases: 7 of them had renal disease (IRIS stages II to IV), 5 had portal hypertension, 4 had pancreatitis, and 1 had liver disease (chronic hepatitis). In 3 cases, the evidence for prescription of omeprazole was considered of equivocal benefit: 2 dogs with hydrocephalus and 1 dog for the prevention of hemorrhage associated with immune-mediated thrombocytopenia. Inappropriate indications for the administration of omeprazole included the prevention of possible NSAID/glucocorticoid-associated GUE in 16 dogs, 5 dogs with non-erosive gastritis, 1 dog with pancreatitis, and 1 dog with renal disease (CKD IRIS II), all of which had no evidence of GI hemorrhage or GUE.

Comparing the two time periods, the percentage of omeprazole prescriptions to treat primary GUE associated with GI disease increased significantly over time (*p* = 0.001). Similarly, there was an increase in the prescription of omeprazole for renal disease associated with GUE (*p* = 0.0072). Conversely, the percentage of cases in which omeprazole was prescribed prophylactically for prevention of possible NSAID/glucocorticoid-associated GUE decreased significantly (*p* = 0.0005). A similar change was observed for cases with non-specific gastritis that was not associated with GUE in which the prescription of omeprazole decreased significantly in 2021 compared to 2017 (*p* = 0.0067). No differences were found between the years 2017 and 2021 for the administration of omeprazole of equivocal benefit (*p* = 0.08).

### 3.4 Associations between different parameters related to omeprazole prescription

When analyzing all data together (2017 and 2021 prescriptions), treatment for ≤4 weeks was associated with prescription q 12 h (*p* < 0.0001) and treatment q 12 h was associated with prescription for the treatment of GI-associated GUE (*p* = 0.0002). On the contrary, treatment q 24 h was associated with prescription of omeprazole for the treatment of non-specific gastritis (*p* = 0.03) and for prevention of NSAID/glucocorticoid-associated GUE (*p* < 0.0001). When each period was evaluated separately, no association was found between the variables in 2017; however, treatment for ≤4 weeks in 2021 was associated with prescription of omeprazole q 12 h (*p* = 0.0003).

### 3.5 Influence of the consensus statement on clinician prescriptions

Sixteen (84.2%) of the 19 clinicians who responded to the survey indicated that they had read the entire consensus statement on prescribing gastroprotectants in dogs and cats (2). Fifteen of them (93.8%) reported that their reading had changed their clinical practice regarding PPIs in dogs.

## 4 Discussion

There are currently a limited number of studies evaluating the prescribing practices of veterinarians using gastroprotectants in dogs and cats (18, 20, 21, 29), and no studies comparing the appropriateness of use of gastroprotectants in dogs and cats prior to and following the publication of the ACVIM consensus statement on the rational use of gastroprotectants in dogs and cats. Approximately 40% of dogs seen in first opinion veterinary practices are prescribed different gastrointestinal agents, including gastroprotectants (22). Similarly, data from referral veterinary hospitals have previously shown that omeprazole is prescribed in around 40% of dogs admitted for hospitalization (20, 21). These studies suggest that similar to what occurs in human medical practice, the administration of PPIs in the hospital setting is of particular concern and may have an impact beyond the hospital stay (20, 21). Administration of PPIs seems to be especially frequent in the hospital setting, in patients with nausea and vomiting, even in the absence of pharmacologic evidence for an antiemetic effect of these drugs (20).

Overall, omeprazole was less frequently prescribed in our hospital (around 10% of cases) than in some previous studies (20, 21), which may be explained by differences in patient demographics, medical training, prescription procedures, hospital caseload, or the proportion of dogs hospitalized to the total number of cases treated. For example, mean ages of the dogs treated in the current study (approximately 10–14 years) was higher than that of animals included in previous studies (5–8 years) (18, 21). In addition, this study included both inpatient and outpatient cases, whereas some of the previously published studies referred only to hospitalized animals (20, 21). Differences may also be explained by the increasing awareness of the rational use of gastroprotectants. Although a lower prescription rate was documented in the first phase of the study compared with the rates described in other studies (20, 21), the significant decrease in prescription of omeprazole over time may support the hypothesis of the increased awareness of the importance of judiciously prescribing PPIs in our hospital. This rate is also lower than the dispensing prevalence of PPIs in the human population in our country, where the use of gastroprotectants, especially in the elderly population, is a public health issue (6).

Previous studies show that 4 and 22% of cases hospitalized in medicine and surgery wards, respectively, received doses of omeprazole that were outside current recommendations (20). In our study, all dogs received therapeutic doses of omeprazole; however, most dogs only received once daily dosing during the first period of the study. There is convincing evidence in the peer-reviewed veterinary literature documenting the suboptimal 24-h gastric acid suppression when PPIs are administered once daily (30–32), and the importance of administering PPIs twice daily was highlighted in the ACVIM consensus statement (2). A significant improvement in the appropriate frequency of administration of omeprazole was observed in the second study period, which occurred approximately 2 years after publication of the consensus statement. The frequency of administration of omeprazole q 24 h was statistically associated with its use for indications for which there is limited scientific evidence documenting the benefits of acid suppression, whereas administration q 12 h was associated with management of disorders in which the beneficial effects of PPI therapy have been well-documented. These statistical associations imply that, within the studied context, clinicians were more inclined to prescribe omeprazole at an incorrect frequency for disorders with less substantial scientific evidence supporting the efficacy of acid suppression. This could stem from a perception that milder or less well-defined disorders may require less intensive treatment.

These results can also be interpreted as a reflection of the awareness of the current scientific evidence on the administration of acid supressants. A 2021 analysis of current trends in prescribing gastrointestinal (GI) protectants among small animal general practitioners revealed that 86 out of 124 respondents (69.3%) reported the practice of administering omeprazole once daily. However, the proportion of general practitioners that recommended once-daily administration of omeprazole was higher than the proportion of those who had no knowledge of the ACVIM consensus statement (29). Twice-daily administration of omeprazole approaches the potential therapeutic efficacy for acid-related diseases when assessed by criteria used for human patients, but median pH, % of time intragastric pH was ≥3, and % of time intragastric pH was ≥4 were all significantly higher when food was withheld than when dogs were fed (30). The ACVIM consensus statement recommends that PPIs should be optimally administered shortly before meals (e.g., 30–45 min) (2) which can be challenging for owners in some cases. Unfortunately, information on the timing of the administration of omeprazole in relation to mealtimes could not be analyzed in our study because written owner instructions were not routinely recorded by staff in the database.

A significant improvement in number of recommendations to gradually taper the omeprazole for prescriptions lasting ≥ 4 weeks was noted after the publication of the ACVIM consensus statement. Some owners were reluctant to follow the taper instructions due to subjective worsening of the dog’s clinical signs in the first few days. A placebo effect could not be excluded in these cases. Rebound gastric acid hypersecretion has been associated with abrupt discontinuation of omeprazole treatment (33), and a tapered approach is recommended for acid suppressants, particularly PPIs, in dogs, cats, and people (2, 33, 34).

Consistent with our study, a remarkable high frequency of inappropriate utilization and administration of omeprazole for a reason unrelated to its expected effects has been previously reported (18, 20, 21). The significant increase in appropriate prescriptions of omeprazole detected in our hospital after the publication of the ACVIM guidelines suggests an alignment with the current scientific evidence, probably based on a philosophy of continuing education and as expected in an academic context. In fact, most clinicians in our study were aware of the consensus statement (2) and they reported that their reading had modified their prescription practices. This included a significant decrease in the prescription of omeprazole for the prevention of NSAID/glucocorticoid-associated GUE and of non-erosive gastritis, although these were still the two most common inappropriate prescriptions in the second study period. An interesting study previously conducted at another veterinary teaching hospital showed a similar trend when comparing prescribing habits retrospectively and prospectively after a clinical audit focused on omeprazole prescription (18). The authors suggested that dissemination of guidelines based on clinical audit may improve prescribing habits (18). In the current study, all data from the two study periods (before and after the ACVIM consensus statement publication) were collected retrospectively, and no specific clinical audit was conducted in the hospital in between, although the ACVIM consensus statement was discussed in journal clubs and during seminars of multiple clinical services at the time of publication.

The prophylactic use of omeprazole in combination with NSAIDs is often considered in practice to be a benign treatment that is more likely to be helpful than harmful (35). However, the scientific evidence does not currently support this recommendation (2, 10, 36) and the co-administration of PPIs with NSAIDs may in fact increase the risk of NSAID-induced intestinal injury by inducing intestinal dysbiosis in people (37) and in dogs treated with piroxicam (35). The exact mechanism for this is not well understood, but the effects of PPIs on the gastrointestinal microbiome contributing to increased cytotoxicity of bile and exacerbation of mucosal injury have been postulated (38). Yoshihara et al. reported that lansoprazole increased the relative abundance of the phylum Bacteroidetes and reduced the thickness of the jejunum mucus layer and globet cells, thereby promoting damage to the small intestine (39). Lastly, the potential alteration of NSAID pharmacokinetics when co-administered with gastric acid suppressants should also be considered (35).

Similarly, corticosteroid administration in dogs significantly increases the incidence of GUE (12, 40, 41). There is limited evidence that acid suppressant drugs are beneficial in preventing GUE when high-dose corticosteroids are used in dogs (2), but their use is common in clinical practice (29). However, a recent study in healthy dogs revealed that the co-administration of omeprazole with prednisone administered at a dose of 2mg/kg q24h partially mitigated hemorrhage in the dogs despite an increased frequency of diarrhea (12). Further evaluation of the protective effects of PPIs on corticosteroid-associated GUE and hemorrhage is warranted in dogs with intestinal disease.

Administration of omeprazole for the management of non-erosive gastritis in dogs is also a frequent phenomenon (18, 20, 21 29), even in the absence of scientific evidence (2). Some authors have mentioned that this practice may have been intended to prevent the possible development of esophagitis, even though this complication seems to be relatively uncommon with intermittent vomiting (20). In our study, the number of omeprazole prescriptions for non-erosive gastritis was significantly lower in the second study period. In most of these cases, the medical records of the affected animals suggested that they were typically severe cases requiring management with multiple medications. The definitive diagnosis or follow-up of these cases was often hindered by the owner’s reluctance to hospitalize their dog or provide consent for diagnostic testing, primarily due to financial constraints.

Although one of the aims of this study was to assess the overall appropriateness of prescriptions, significant changes in the relative frequency of specific indications for omeprazole prescription were detected. It is interesting to note that appropriate prescriptions of omeprazole (e.g., GUE or renal disease with intestinal hemorrhage) increased significantly over time, whereas inappropriate prescriptions (e.g., non-specific gastritis or prophylaxis for NSAID/glucocorticoid administration) decreased. Although the most likely hypothesis is that these changes were due to improved prescribing, it cannot be excluded that these results may have been influenced by potential differences in the caseload and frequency of presentation of the different diseases in the hospital, a phenomenon that was not evaluated.

There were several other limitations of this study, including its retrospective nature and the inherent lack of sufficient detail on drug dosing, administration recommendations in relation to mealtimes, and guidelines on tapering the drug following prolonged administration. As physicians’ experience and demographic factors, such as age and gender, have been shown to significantly influence the prescription of certain drugs, it would have been interesting to determine the impact of the ACVIM consensus statement on each individual level of training or expertise of the veterinarians (interns vs. residents vs. faculty) involved in managing the cases during the first and second periods of study. However, demographic data were not collected and the prescribing practices of interns and residents may have been impacted by guidance and mentoring of faculty on service in this setting. The results of our study should be interpreted with caution as they may not necessarily reflect the situation in first-opinion practices or in other teaching hospitals.

The significant increase in appropriate omeprazole prescribing practices following the publication of the 2018 ACVIM consensus statement underscores the positive impact of evidence-based guidelines on shaping veterinary practice. Consensus statements may not only improve the quality of patient care but may also address broader societal concerns related to responsible and judicious medication use in veterinary medicine. The findings from this study and others emphasize the importance of ongoing education and awareness campaigns to further improve prescribing practices across the profession.

In conclusion, this study found a significant increase in the frequency of appropriate omeprazole prescriptions following the publication of the 2018 ACVIM consensus statement, in the absence of specific improvement interventions such as a clinical audit. Further efforts should be made to improve remaining inappropriate prescriptions in clinical practice. In addition, further studies are needed to clarify the potential efficacy of acid suppressants in clinical situations where scientific evidence is currently still limited.

## Data availability statement

The original contributions presented in the study are included in the article/supplementary material, further inquiries can be directed to the corresponding author.

## Ethics statement

The studies involving human participants were reviewed and approved by the Institutional Review Board of the Complutense Veterinary Teaching Hospital.

## Author contributions

ÁS: Conceptualization, Data curation, Methodology, Writing – original draft. MG-S: Data curation, Writing – review & editing. AV: Data curation, Writing – review & editing. FR-F: Data curation, Writing – review & editing. DD-R: Data curation, Writing – review & editing. PO: Data curation, Writing – review & editing. SM: Conceptualization, Methodology, Writing – original draft.
